# Ukrainian healthcare providers under siege during the first year of war: challenges and adaptations

**DOI:** 10.1192/bji.2023.43

**Published:** 2024-05

**Authors:** Alexandra A. Deac, Irina Zaviryukha, Oleksandr Zeziulin, Anna Peycheva, Renata Solórzano de Souza, Harry Skipper, Asmau Abubakar, V. Benjamin Gustilo, Sheela V. Shenoi, Graham Thornicroft, Julia Rozanova

**Affiliations:** 1 MSc, Research Affiliate, Department of Health Service and Population Research Department, King's College London, London, UK; 2 MD, Researcher, European Institute of Public Health Policy, Kyiv, Ukraine; 3 MPhil, Senior Researcher, European Institute of Public Health Policy, Kyiv, Ukraine; 4 MSc, Research Affiliate, Department of Health Service and Population Research Department, King's College London, London, UK; 5 MSc, Student, Department of Health Service and Population Research Department, King's College London, London, UK; 6 MSc, Student, Department of Psychological Medicine, King's College London, London, UK; 7 MSc, Student, Department of Health Service and Population Research Department, King's College London, London, UK; 8 MSc, Student, Department of Psychological Medicine, King's College London, London, UK; 9 MPH, Associate Professor of Medicine, Section of Infectious Diseases, Yale University School of Medicine, New Haven, Connecticut, USA; 10 PhD, Professor of Community Psychiatry, Centre for Global Mental Health, Institute of Psychiatry, Psychology and Neuroscience, King's College London, London, UK; 11 PhD, Lecturer in Global Mental Health, Department of Health Service and Population Research Department, King's College London, London, UK. Email julia.rozanova@kcl.ac.uk

**Keywords:** Healthcare providers, Ukraine, mental health, system adaptations, system challenges

## Abstract

The overlapping COVID-19 crisis and the war starting in 2022 threaten front-line healthcare workers’ mental health, well-being and job retention in Ukraine. This paper provides a synopsis of a panel discussion held by the Global Mental Health Humanitarian Coalition in May 2022 and expert consultations with clinicians between December 2022 and February 2023 on these challenges. The crises created new problems and exacerbated many pre-existing difficulties. We found that healthcare workers had needed to mobilise previously untapped strengths, including portable emergency medical documents and bespoke local psychosocial support services, amid the costs and pressures of ongoing healthcare reforms.

The Russian invasion of Ukraine on 24 February 2022 led to significant disruptions and damage to the country, with almost 14 million people being internally displaced or forced to flee as refugees.^1^ The healthcare infrastructure was a target in the early months of conflict, necessitating urgent adaptations to maintain a viable health service. This paper draws on: (a) a panel discussion held by the Global Mental Health Humanitarian Coalition (GMHHC)^2^ and (b) face-to-face and telephone expert consultations with administrators, doctors, nurses, psychologists and social workers from Kyiv City AIDS Centre, the Narcological Clinical Hospital ‘Sociotherapy’ and Kramatorsk Narcological Dispensary (Donetsk region), between December 2022 and February 2023. This paper aims to identify the impact of war on mental health and on-the-ground mental health services. The information sources are staff who were pragmatically available to be consulted, working in HIV/AIDS departments and addiction treatment centres; however, these insights tap into the experience of the broader healthcare system.

## The main mental health and mental healthcare challenges identified

The concurrence of the COVID-19 crisis and war with Russia poses an existential threat to front-line healthcare workers’ lives, health and mental health in Ukraine. The GMHHC panellists identified a series of major challenges to the healthcare infrastructure in Ukraine. Initially, these services had been threatened with collapse due to conflict escalation and the heavy toll on front-line workers’ mental health as they had to intensify their efforts to meet the increasing numbers of patients.^2^ It is clear that Ukrainian healthcare staff are facing an increased workload, particularly as the displaced population moved within the country or abroad to flee the war.^3^ For the general population, World Health Organization (WHO) has estimated that nearly 10 million people in Ukraine might have at least one type of mental disorder following direct or indirect exposure to war.^4^ Healthcare staff also experience adverse mental health outcomes, such as post-traumatic stress disorder (PTSD), depression, anxiety and substance use disorders, and face extreme challenges in trying to fulfil their professional duties and maintain a decent quality of patient care.

## The resourcefulness of healthcare staff

Despite direct attacks on healthcare facilities by Russian military forces and their efforts to block the delivery of humanitarian aid,^5^ healthcare staff are showing remarkable resourcefulness and resilience.

Three months after the full-scale Russian invasion, the GMHHC panel identified that there had been a rapid implementation in the online delivery of psychological first aid, aimed mainly at the general population. Expert consultations revealed that this collective effort was made proudly and was seen as epitomising a moral victory among the Ukrainian staff. Study informants emphasised that healthcare staff in Ukraine have gone above and beyond their traditional duties. For example, some clinicians prepared emergency document summaries from medical records (e.g. letters of diagnosis and treatment, prescriptions) and distributed them to fleeing patients to take with them to ensure continuity of their care. These emergency records also allowed staff to receive contractual compensation for their clinical work, especially in treating displaced persons.

Because of the impact of war, healthcare staff themselves quickly became a vulnerable group owing to heightened stress, long working hours, anxiety, poor sleep and the increased scarcity of essential supplies. This required specific support for mental health staff. For example, during our winter expert consultations of 2022–2023, Ukrainian colleagues on the ground described tailoring counselling services to staff within their clinics.

## The wider context of healthcare reform in Ukraine

Healthcare reforms in Ukraine in recent years, such as the 2015 progress towards the National Health Service of Ukraine (NHSU)^6^ and the financial reform and governance of the Program of Medical Guarantees (PMG),^7^ significantly strengthened the healthcare system. For example, the PMG covers in-patient care which, in turn, reduces the out-of-pocket costs for patients, and the NHSU moved specialised care services to primary health care (PHC). When the COVID-19 pandemic hit, the PMG expanded to a total of 31 packages of care, which included a COVID-19 salary top-up for healthcare providers, with varying rates for doctors (70% of salary rate), nurses (50%) and junior medical doctors (30%), and continued throughout the war.^7^ Consequently, PHC contractual coverage was nearly 70% by 2021, while specialised care, including psychiatric and addiction units, suffered considerable staffing reductions due to PHC task-shifting.^8,9^

Two years into the conflict with Russia, the Ukrainian health system still features universal coverage,^10^ so that money follows patients. These reforms are cost-effective and can lead to job enrichment among staff remaining in those roles; nevertheless, such instability in the healthcare system threatens sustainability when healthcare providers are ‘not only fired (from their jobs) but also fired upon’, according to our study participants.

The study informants also raised the challenges of staff who were given extra responsibilities during the conflict, which limited their own freedom to flee the war or conflict zones for fear of losing their jobs. Similarly, the conflict in Ukraine has resulted in the redirection of resources, shifting priorities and funding channels within the healthcare sector, with grave implications for healthcare infrastructure, facilities and resources. Although many international organisations have provided financial relief to the Ukrainian health responses, in Spring 2023 a WHO appeal indicated a gap of 77% (US$184.2 million) for healthcare needs.^11^ A further serious challenge for staff is that described as ‘moral injury’, which occurs when staff need to make vital decisions about the prioritisation or allocation of healthcare resources, often with immediate and grave effects for patients, with little or no training or support.

## Lessons learned

These insights lead us to the following lessons. First, at the individual and local levels, we have learned that healthcare staff in Ukraine became ‘volunteering martyrs’, driven by their love for their profession and love of their country. For many, contributing their professional services was a way to express and demonstrate their patriotism during the war. Although the risks of emotional exhaustion, compassion fatigue, burnout and adverse mental health outcomes among healthcare workers can lead to decreased motivation or even failure of the healthcare system, valuable lessons must be learned from these challenges. Along with cost-effective solutions, strategies such as a managed workload distribution and fair recognition of staff contributions can maintain a motivated workforce. Similarly, determination and commitment can drive individuals to demonstrate remarkable resourcefulness in developing, adapting and managing very limited resources. For instance, expanding on the availability and acceptability of telehealth in Ukraine towards co-producing and tailoring mental health courses, seminars, career advancement opportunities or sustainable capacity-building programmes strengthened this sense of community, normality and stability among healthcare staff.

Second, the healthcare workforce, including mental health counsellors and those working at the intersection of addictions, HIV/AIDS and other chronic stigmatised conditions, have never received sufficient resources compared with other sectors of government, while the demands on healthcare personnel have grown exponentially during the war. Incorporating the needs of healthcare workers into policy priorities is essential to prevent system collapse and ensure the continuous provision of critically important services. In addition, the workplace needs to be seen as a place of collaboration that prioritises the mental health and well-being of both patients and staff. Active elements could feature more professional autonomy, including the ability to develop or invest in local programmes and initiatives, decentralisation of care and strengthened partnerships between agencies. At the same time, such approaches require participatory initiatives to empower meaningful bottom-up solutions that can help retain skilled staff, protect the overall impact on the population's health and prevent an exodus of healthcare staff.

The results of these consultations showed that the statement ‘No one is safe until everyone is safe’ in global and mental health is now more pertinent than ever.^12^ These are very difficult problems, and the solutions are complex, starting from local clinic managers engaging in open and transparent collaboration with various stakeholders, including donors, policymakers, patients and governmental ministries. International partners and organisations who wish to assist Ukraine must prioritise mental health as part of their investment and support packages. Leaving the strengthening of mental health infrastructure until after the war will be too late. It is critical to invite mental healthcare staff into the joint planning of relief efforts democratically and inclusively, leveraging their insights and first-hand knowledge regarding contextual complexities. Additionally, mental health staff need to be included in preparedness efforts such as emergency simulations to enhance intersectoral collaboration and shock absorption as disasters unfold. Therefore, these insights demonstrate a need for well-orchestrated contextual policies, integrated national and international efforts and sustainable workforce planning.

## Data Availability

The published materials that support the findings of this study are openly available in the referenced literature. The data from studies conducted in Ukraine by Dr Rozanova and colleagues that support the findings of this study are available from the corresponding author, J.R., upon reasonable request.
